# Captive Rearing Experiments Confirm Song Development without Learning in a Tracheophone Suboscine Bird

**DOI:** 10.1371/journal.pone.0095746

**Published:** 2014-04-30

**Authors:** Janeene M. Touchton, Nathalie Seddon, Joseph A. Tobias

**Affiliations:** 1 Smithsonian Tropical Research Institute, Balboa, Panama; 2 Max Planck Institute for Ornithology, Radolfzell, Germany; 3 Edward Grey Institute, Department of Zoology, University of Oxford, Oxford, United Kingdom; UCLA, United States of America

## Abstract

The origin of vocal learning in animals has long been the subject of debate, but progress has been limited by uncertainty regarding the distribution of learning mechanisms across the tree of life, even for model systems such as birdsong. In particular, the importance of learning is well known in oscine songbirds, but disputed in suboscines. Members of this diverse group (∼1150 species) are generally assumed not to learn their songs, but empirical evidence is scarce, with previous studies restricted to the bronchophone (non-tracheophone) clade. Here, we conduct the first experimental study of song development in a tracheophone suboscine bird by rearing spotted antbird (*Hylophylax naevioides*) chicks in soundproofed aviaries. Individuals were raised either in silence with no tutor or exposed to standardized playback of a heterospecific tutor. All individuals surviving to maturity took a minimum of 79 days to produce a crystallized version of adult song, which in all cases was indistinguishable from wild song types of their own species. These first insights into song development in tracheophone suboscines suggest that adult songs are innate rather than learnt. Given that empirical evidence for song learning in suboscines is restricted to polygamous and lek-mating species, whereas tracheophone suboscines are mainly monogamous with long-term social bonds, our results are consistent with the view that sexual selection promotes song learning in birds.

## Introduction

Vocal learning – the encoding and production of acoustic traits acquired from conspecific or heterospecific tutors – is restricted to very few groups of birds and mammals, and understanding its origins remains a core aim of evolutionary biology [1], [2]. One of the primary study systems for exploring the adaptive significance of vocal learning is birdsong, yet the distribution of vocal learning in birds is contentious. The prevailing view is that song learning is restricted to three major clades, the oscine passerines (Passeriformes), parrots (Psittaciformes), and hummingbirds (Trochiliformes). In contrast, it is often assumed that songs develop without learning from tutor birds in the suboscines, a diverse clade of passerines containing ∼1150 species. However, the finding that parrots are the sister group to both clades of passerines, the oscines and suboscines [3], [4], has cast some doubt on whether suboscines develop songs by learning or not [2], [5], [6]. In addition, recent evidence that vocal learning occurs in some species of suboscine suggests that they offer a better system for understanding the origins of this trait than do oscines and parrots, wherein learning is virtually universal.

Previous experimental tests of vocal learning in suboscines have shown that Willow (*Empidonax traillii*) and Alder (*Empidonax alnorum*) flycatchers raised with heterospecific tape tutors [7], and deafened Eastern Phoebes (*Sayornis phoebe*) [8], all produce normal adult song. These species also lack the forebrain cell clusters that control song acquisition in oscines [8], although recent studies have shown that at least one of these species, *S. phoebe*, has an incipient or vestigial homologue of these cell clusters [2]. It was often inferred on the basis of these studies that suboscines were unable to learn their songs, but this paradigm has recently been challenged. For example, several lines of evidence suggest that *Procnias* bellbirds develop songs with learning [5], [9]: (1) mismatch between genetic variation and geographic variation in male songs of *Procnias tricarunculata*; (2) intricate changes in song over time within individuals; (3) the production of heterospecific song of a cage mate by a captive *Procnias nudicollis*; and (4) the production of bilingual, rather than hybrid dialects. Vocal learning has also been proposed for the long-tailed manakin *Chiroxiphia linearis* [10], [11] and screaming piha *Lipaugus vociferans* [12], although in both cases the effects of learning appear to be weak, and further supporting evidence is required [13], [14].

These studies have led to growing claims that vocal learning may be widespread in suboscine passerines, although all research so far has focused on tyrant-flycatchers (Tyrannidae), manakins (Pipridae) and cotingas (Cotingidae) belonging to the infraorder Tyrannides, sometimes referred to as the non-tracheophone (or “bronchophone”) suboscines [15]. In contrast, almost no attention has focused on the infraorder Furnariides – i.e. tracheophone families such as the antbirds (Thamnophilidae) and ovenbirds (Furnariidae) – that make up about half of suboscine species (and approximately 5% of total global bird diversity). Consequently, the widespread assumption that vocal learning is absent in the tracheophone clade [16]–[18] is based entirely on inference or anecdotal observations.

Direct experiments in tracheophones are clearly a priority, particularly as they may shed light on the role of social mechanisms, including sexual selection, in the evolution of vocal learning. Sexual selection has been proposed to explain variation in learning across oscine passerines, although evidence is contradictory and widespread support is lacking [19]–[23]. Some oscines subject to strong sexual selection have large song repertoires [24] and are often capable of sophisticated mimicry [25], while others place a higher premium on vocal traits unrelated to learning, such as performance consistency [26]. The link between sexual selection and vocal learning may be easier to detect in suboscines, where all species proposed to learn songs have lek-based or polygamous reproductive strategies, and thus intense sexual selection. This includes cases of learning in bellbirds [5], [9], manakins [10], [11] and pihas [12]. In contrast, the sexual selection hypothesis predicts that learning will be rare or absent in tracheophones as all members of this clade have long-term social bonds and thus apparent low levels of sexual selection [27]. However, experiments testing the mode of song development are still lacking in this major branch of the suboscine radiation.

To address this issue, we raised spotted antbirds (*Hylophylax naevioides*) by hand from the egg in soundproofed aviaries, exposing developing chicks to either silence (no tutor), or the song of a congeneric species from Amazonia, *Hylophylax naevius* (heterospecific tutor). This species was an ideal tutor as it is closely related to *H. naevioides*, with a song that is essentially similar in overall tone and pattern, but with a distinctly different cadence that can easily be detected in acoustic analyses. Our aims were two-fold: (1) to quantify the progression of song development in captive-reared birds from hatching to the production of adult song, and (2) to compare the structure of the adult songs of captive-reared birds in our two treatment groups with those of wild conspecifics and heterospecific tutors.

## Materials and Methods

### Ethical statement

This study was approved by and performed in accordance to The Smithsonian Tropical Research Institute Institutional Animal Care and Use Committee (IACUC # 2009-01-03-17-09). Additionally, field protocols were approved by and performed in accordance to the Autoridad Nacional del Ambiente (ANAM) of Panama (# SE/A-61-09).

### Study species

**Figure 1:**
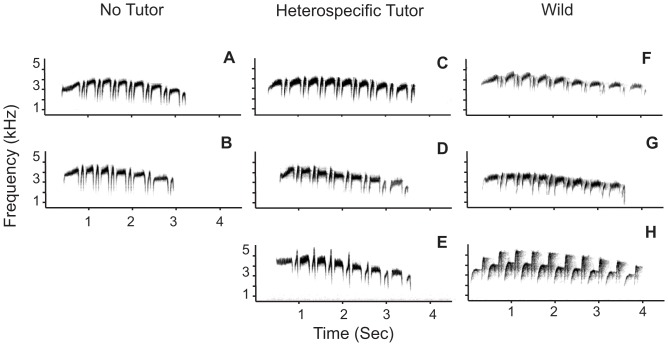
Representative song spectrograms from individuals in each treatment group . Broadband spectrograms show (**A, B**) captive *H. naevioides* reared in silence with no tutor, (**C–E**) captive *H. naevioides* reared with *H. naevius* tutor, (**F**) wild male *H. naevioides*, (**G**) wild female *H. naevioides*, (**H**) wild male *H. naevius*.

*H. naevioides* is a tracheophone suboscine passerine found in Central America, north Colombia, and Ecuador. Monogamous pairs form long-term pairbonds where both sexes contribute equally to parental care and defence of stable year-round territories [28], [29]. Both sexes sing a structurally similar stereotyped song (Figure 1F, 1G) [30]. Consistent with other antbirds [31], [32], these songs are individually recognizable [30] and given year-round with a slight peak in vocal activity in the breeding season, suggesting that they mediate competition over both mates and territories [33].

### Collection and bird handling

We conducted collection efforts from April to June, 2009–2010, near Soberanía National Park, in the vicinity of Gamboa, Panamá (9°7′N, 79°40′W). As nestling passerine birds can potentially imprint on the songs of adults immediately after hatching [34], we collected study individuals of *H. naevioides* at the egg stage. A total of 32 eggs were removed from 16 nests with complete clutches (2 eggs) in the early breeding season. This ensured that siblings could be divided among treatments (see below), and that adult pairs would readily re-nest, minimizing impacts on the population.

Within 1 h of collection, we placed eggs in a Grumbach Compact S84 incubator with automatic temperature and humidity set at 36.5°C and 70%, respectively, based on field data collected from active *H. naevioides* nests (Gustavo Londoño, *unpublished data*). Eggs were automatically turned every hour throughout the duration of incubation. One day prior to hatching, we placed eggs in individual tissue-lined nest cups in a Brinsea TLC-4 brooder (temperature: 36°C; humidity: 70–80%). Over the course of 11 days – i.e. the mean time for chicks to fledge their nest in the wild [28], [35] – we gradually lowered temperatures in brooders to 28°C (experimental room temperature).

Day 1 after hatching we hand-provisioned chicks with 60–75% of their body mass in lab-reared waxworms and crickets, augmenting this diet with small wild-caught katydids. At day 2, we syringe-provisioned chicks with FoNS (formula for nestling songbirds) gruel [36], [37] and insects as above every 30–60 minutes depending on intake quantity. Feed rate was adjusted to ensure a daily intake of 60–75% of the chick's body mass up to day 8, rising to 50–60% of body mass at fledgling (approximately day 11). Chicks were weighed every morning (06:30 h) and evening (18:00 h), and following each feeding. Average weight gain and developmental rates were similar to those of wild nestlings [28].

Upon fledging, we moved chicks to individually isolated 84 cm×46 cm×76 cm flight cages where they were provided *ad libitum* with a balanced adult diet (mashed soaked EVO cat food, boiled egg, bird vitamins, yoghurt, and live mealworms and crickets). We gradually weaned chicks off FoNS gruel until they demonstrated independent feeding of this mash from self-feed pans. We measured the quantity of food consumed by individuals to ensure a sufficient daily intake of 40–50% of the body mass to maintain a mass of 16–20 g. We changed water twice daily, dropping mats daily, and cleaned perches and cages weekly.

Health was assessed throughout the study by weekly faecal analysis and daily visual inspection for signs of illness (feather expansion, extended eyelid closure, and lethargy). Of 14 eggs collected in 2009, all successfully hatched, 5 fledged, 5 survived to maturity, and 5 completed the experiment in a healthy condition. In 2010, 17 of the 18 eggs successfully hatched, but none completed the experiment due to recurring problems with coccidial infection at the pre-fledgling developmental stage. Given the good health of the individuals exposed to experimental treatments, and their vigorous singing behaviour, we believe that our results are not affected by any challenging physical condition.

### Experimental Procedure

At hatching, we randomly assigned individuals to one of two treatment groups: (i) no tutor (i.e., silence) or (ii) heterospecific tutor. Because treatments began seven days after hatching, at which point it is not possible to sex individuals accurately, we assigned treatment groups independently of sex. Of the five individuals reaching maturity, two were males in the no tutor group, and three were females in the heterospecific tutor group. Prior to fledging, we placed individuals in sound attenuation chambers only during their daily tutor sessions, whereas after fledging they were permanently housed individually in flight cages within sound attenuation chambers (see below) for the duration of the experiment.

**Figure 2:**
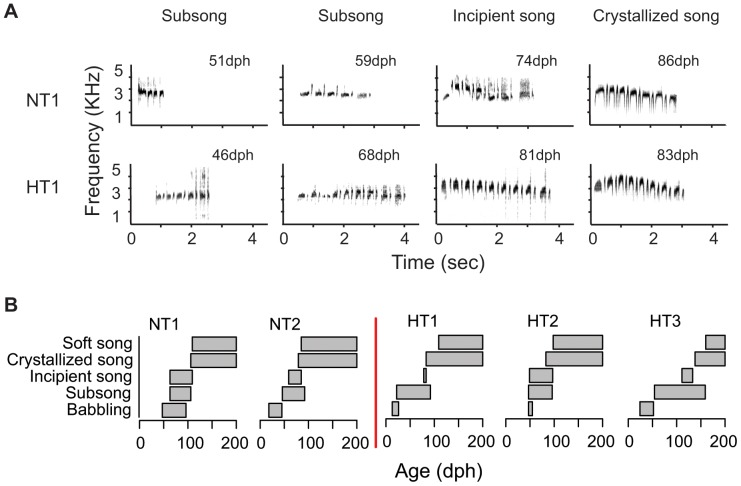
Development of song in *Hylophylax naevioides*. (**A**) Broadband spectrograms of vocalizations produced by two captive individuals demonstrating different stages towards the production of crystallized adult song. Upper panel, a male bird reared in isolation with no tutor (NT1): subsong at 51 and 59 days post hatching (dph); incipient song at 74 dph; crystallized song at 86 dph. Lower panel, a female bird reared in isolation with a heterospecific tutor (HT1): subsong at 46 and 68 dph; incipient song at 81 dph; crystallized song at 83 dph. (**B**) Ages (dph) at which the five stages of song production (babbling, subsong, incipient song, crystallized song, and soft song) were produced by two male birds reared in isolation with no tutor (NT1, NT2; left of the red line) and three female birds reared in isolation with a heterospecific tutor (HT1, HT2, HT3; right of the red line), until the end of the experiment for each individual (200 dph). See methods for description of song production developmental stages.

Tutor sessions began daily at 07:00 h and lasted 1 h. The no-tutor group received silence; the heterospecific tutor group received playback of *H. naevius* song. To track song development through to the production of adult song, we recorded all individual birds daily from 10 minutes prior to – 1 h after tutor sessions. All digital sound files were 16 bit wav mono files recorded at a sampling frequency of 44.1 kHz. The experimental treatment concluded when individuals had produced a minimum of 10 high quality adult songs (between 83 and 165 days; see Fig. 2B).

### Experimental set-up

#### Sound attenuation chambers

We constructed sound attenuation chambers using a double wall design and open cell polyether polyurethane acoustic foam coated with a moisture and chemically resistant film (Soundcoat soundfoam M with uniseal). This design provides sound attenuation levels of approximately 30 dB. Chambers were ventilated with silicone tubing attached to an air pump and equipped with full-spectrum fluorescent light strips programmed to mimic diurnal cycles in the wild. To record vocalizations, chambers were equipped with cardioid condenser hanging microphones (Audio-Technica U853A) connected to external high-resolution Solid State wav recorders (Edirol R-09HR). Chambers in the heterospecific tutor treatment group were additionally equipped with mini-amp speakers (Radioshack). Chambers were located in two separate climate controlled experimental rooms, one for each treatment group. Treatment groups were split between experimental rooms to avoid the potential risk of individuals hearing vocalizations during routine husbandry.

#### Playback treatment preparation

We used RavenPro version 1.4 to prepare playback audio (wav) files for the heterospecific tutor group. Playback loops consisted of 10 *H. naevius* songs separated by 3 sec of silence, followed by 5 min of silence, similar to natural rates of singing in the wild. To avoid pseudoreplication, we used unique playback loops from recordings of songs from different individuals of *H. naevius* for each individual in the heterospecific tutor group.

### Sampling songs of wild individuals

To compare the songs of captive-reared individuals with those of wild conspecifics (*H. naevioides*), we recorded 6 high-quality songs from both males (N = 15) and females (N = 17) in Soberanía National Park. Recordings were made between September 2009 and February 2012 between 06:30 h and 11:00 h. Songs were recorded on compact flash cards as 16-bit wav mono files at a sampling frequency of 44.1 kHz using a Sennheiser ME67-K3U directional microphone (Sennheiser, Hanover, Germany) and Marantz PMD-661 Solid State recorder (Marantz, Kanagawa, Japan). We solicited singing on territories using playback (prepared as above) of male or female songs.

We also compiled recordings of wild heterospecific (*H. naevius*) individuals, with songs of 11 unsexed birds downloaded from a digital archive (http://www.xeno-canto.org/; file accession numbers: XC63522, XC63523, XC90280, XC44136, XC74917, XC39754, XC33214, XC2993, XC90289, XC3724, XC98060) and a further individual extracted from a commercial CD (N = 1; [38]).

### Acoustic analysis

**Figure 3:**
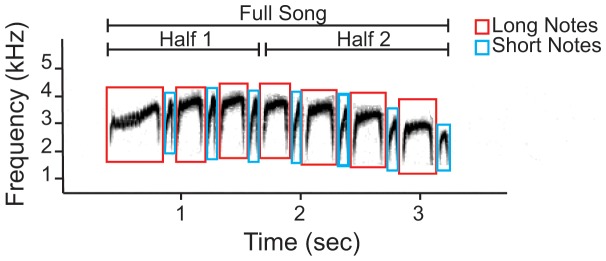
Acoustic analysis of *Hylophylax naevioides* song. Broadband spectrogram illustrates an example of one song produced by a wild adult male. Boxes denote the acoustic selections used in this study to calculate acoustic parameters using robust statistical estimators in Raven 1.4. Parameters were calculated as averages across five different subsets of notes separately: the full song, the first half of the song by the nearest note to the middle time, the second half of the song by the nearest note to the middle time, the long notes and the short notes (see Table 1).

Using RavenPro version 1.4, we produced broadband spectrograms (bandwidth = 61.9 Hz, Hann window size = 1024) of songs of all study individuals, to compare songs produced by captive-reared birds with those of wild *H. naevioides* and *H. naevius*. Assessment was conducted qualitatively by visual inspection, and quantitatively by extracting acoustic parameters by hand from spectrograms using on-screen cursors (see Fig. 3). This technique is sensitive to errors generated by small changes in the exact placement of on-screen selections, and environmental background noise. We minimized such errors using RavenPro to generate a set of robust statistical estimators for each selection. This approach reduces the effect of outliers by using measures of central tendency and dispersion such as order statistics, the median, interquartile range, and quartile skewness (see [39]). We used 4 spectral and 4 temporal robust estimators to quantify a total of 19 acoustic parameters for each song (see Table 1). By nature of having multiple elements within songs, long note and short note song parameters per song were generated by averaging robust estimators taken for each long note and short note within an individual song, respectively, prior to further analysis. This step was unnecessary for the other acoustic parameters that only had one element per song (i.e., entire song, first half of song, second half of song).

**Table 1:** Description of acoustic parameters extracted from songs (see Figure 3).

Robust acoustic parameter	Definition	Specific measurement*
Centre Frequency (Hz)	Frequency that divides the selection into two frequency intervals of equal energy†	1. Entire song
		2. 1st half of song
		3. 2nd half of song
		4. Long note
		5. Short note
		6. Difference between 1st and 2nd half of song
1^st^ Quartile Frequency (Hz)	Frequency dividing the selection into two frequency intervals containing 25% and 75% of the energy†	7. Long note
		8. Short note
3^rd^ Quartile Frequency (Hz)	Frequency dividing the selection into two frequency intervals containing 75% and 25% of the energy†	9. Long note
		10. Short note
Inter-quartile Range Bandwidth (Hz)	The difference between the 1^st^ and 3^rd^ Quartile Frequencies	11. Long note
		12. Short note
Centre Time (sec)	The duration the selection is divided into two time intervals of equal energy†	13. Long note
		14. Short note
1^st^ Quartile Time (sec)	The duration that divides the selection into two time intervals containing 25% and 75% of the energy†	15. Long note
		16. Short note
3^rd^ Quartile Time (sec)	The duration that divides the selection into two time intervals containing 75% and 25% of energy†	17. Short note
Inter-quartile Range Duration (sec)	Difference between the 1^st^ and 3^rd^ Quartile time	18. Long note

*For all parameters except 1–3, mean was calculated for notes within a song.

† Power values in short-time spectra and frequency bands that compose the spectrogram are summed to generate aggregate power envelopes and spectra, resulting in a power versus time envelope and power versus frequency spectrum, respectively.

The aggregates are normalized and treated as probability density functions with time or frequency being the variate, and density the fraction of the total signal energy. From the distribution function, various measures of central tendency and dispersion are then used to characterize the signal energy distribution in time and frequency. (See [39]).

### Statistical analysis

To compare songs produced by captive-reared individuals with those of wild birds, we first calculated mean individual values for each song acoustic parameter. We then performed a series of rotated principal components analyses (PCAs) with Kaiser normalization on the correlation matrix of mean values of song parameters to reduce the dimensionality of our dataset and to avoid multicollinearity. Four PCAs were conducted on four different subsets of the dataset: (1) all *H. naevioides* individuals, (2) only male *H. naevioides*, (3) only female *H. naevioides*, and (4) both *H. naevioides* and *H. naevius* (sexes pooled). In each case, PCA extracted three PC scores with eigenvalues >1, which accounted for >85% of variance in the acoustic datasets (Table S1). We then used PC scores (1) to test for differences in the structure of male and female songs, (2) to test for differences between songs produced by captive-reared and wild birds, (3) for a Discriminant Function Analysis (DFA), (4) for a bootstrap test, and (5) to plot the structure of all songs in relation to one another.

We compared the structure of captive and wild antbird songs in three complementary ways. First, we used an ANOVA to compare PC scores between wild and captive songs. Because we found no significant difference in the structure of male and female *H. naevioides* songs as described by all three PCs (Table 2), we pooled song data from the sexes for this analysis. Second, we used DFA with cross-validation using the lda function in the MASS package [40]. We ran the DFA on PC scores calculated as above with the addition of 2 PC scores to account for 95% percent of the total variance in the acoustic datasets. We then calculated the proportion of captive-reared treatment individuals that were grouped with the wild birds, heterospecific tutor birds, or as captive-reared birds based on the PC scores generated from the acoustic song datasets. Third, we determined whether the overall structure of the songs produced by captive-reared birds (as defined by PC1, Table S1) fell within the natural range of acoustic variation we sampled in wild birds. To do this, we randomly selected data for a single individual in the wild-song dataset, extracted the PC1 score, and repeated (allowing the same individual to be selected more than once) until we had generated the same number of data points as the total number of wild individuals sampled (N = 32). We repeated these steps 10,000 times, then plotted means for each bootstrap replicate, and finally assessed whether the mean PC1 scores for captive-reared birds fell within the null distribution of the PC1 scores for the wild birds. Bootstrap tests were conducted on each treatment (and hence sex) separately.

**Table 2:** Effect of captive rearing on song structure in *Hylophylax naevioides*, where song is defined by PC1, PC2 and PC3 (mean ± SD).

Variable	Captive-reared	Wild song	*X^2^* *	*P*
PC1	−0.19±2.57	0.03±3.25	0.01	0.93
PC2	−1.03±1.60	0.16±2.44	1.14	0.29
PC3	−0.59±0.85	0.09±1.04	2.28	0.13

*Statistics derive from a Kruskal Wallis test; N_1_ = 5 captive-reared birds, N_2_ = 32 wild birds (sexes pooled).

## Results

### Song development

The five captive-reared individuals in this study all followed the same progression in vocalization types towards the production of crystallized adult song, although the timing of each stage varied (Fig. 2A, 2B). Three individuals varied only marginally in the timing of the progression to crystallized adult song, between 79 and 83 days post hatching (dph), but this progression was longer for one individual in the no tutor group (106 dph) and one individual in the heterospecific tutor group (138 dph) (Fig. 2). All individuals began ‘babbling’ as their first step in song production between 13 and 47 dph (Fig. 2B). Babbling appeared to be a modified version of the contact call produced by all individuals immediately upon fledging. It took from 1 to 30 days to progress from babbling to the second step, which we term ‘subsong’ (Fig. 2B). Subsong comprised actual components and groups of notes of a complete adult song. In all but one individual (HT3, in the heterospecific tutor group), initial subsong was similar to the middle section of adult song, but without any inflection in pitch. HT3 took longest to progress from the babbling phase, and was unique in including the introductory section of adult song into her subsong. During the subsong phase, individuals either added more notes to sections or produced different parts of adult song – either the introductory, middle, or terminal section – until they were able to produce an ‘incipient adult song’. The delay between the start and end of the subsong phase varied from 1 to 57 days. Incipient adult songs were often missing a rise and fall in pitch and notes lacked the clarity of those produced during ‘crystallized adult song’. All individuals required some time in the incipient adult song phase (15–43 days) before they were able to produce crystallized adult song. Following the production of crystallized song, individuals continued to produce sub and incipient song for a further 3–21 days until they could produce consistent crystallized adult song (Fig. 2B).

### Comparison of adult songs in captive and wild birds

**Figure 4:**
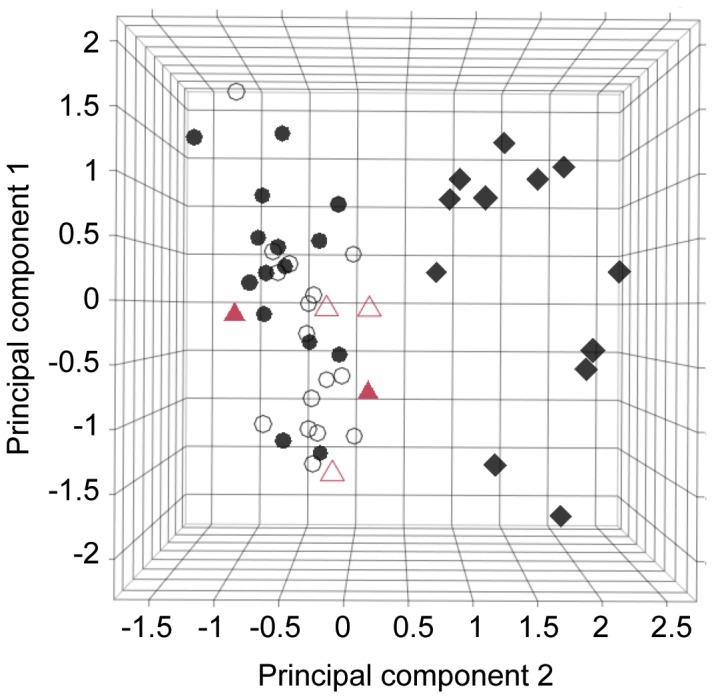
Comparison of the structure of the songs of experimental versus wild individuals. Songs by male (closed symbols) and female (open symbols) wild (circles) and captive-reared (red triangles) *H. naevioides* grouped together while those produced by *H. naevius* (closed diamonds) grouped separately. Plot produced according to three principal components generated from acoustic data extracted from spectrograms; PC3 is represented by depth and is not labelled (see Table S1 for factor loadings).

Visual inspection of spectrograms of crystallized adult songs produced by captive-reared *H. naevioides* showed them to be extremely similar in spectral structure and temporal patterning to those produced by wild conspecific adults (Fig. 1). Accordingly, when we plotted the structure of the songs of all individuals in our sample in the acoustic space defined by PC1, PC2 and PC3, we found that captive-reared birds produced songs that fell within the area of acoustic space occupied by songs produced by wild *H. naevioides* (Fig. 4). Specifically, the songs of the two individuals raised in silence with no tutor fell within the area occupied by wild conspecific adults, as did the songs produced by the three individuals raised in the heterospecific tutor group. These qualitative appraisals of the similarity of songs of captive individuals to the songs of wild conspecifics were corroborated quantitatively in three ways.

**Figure 5:**
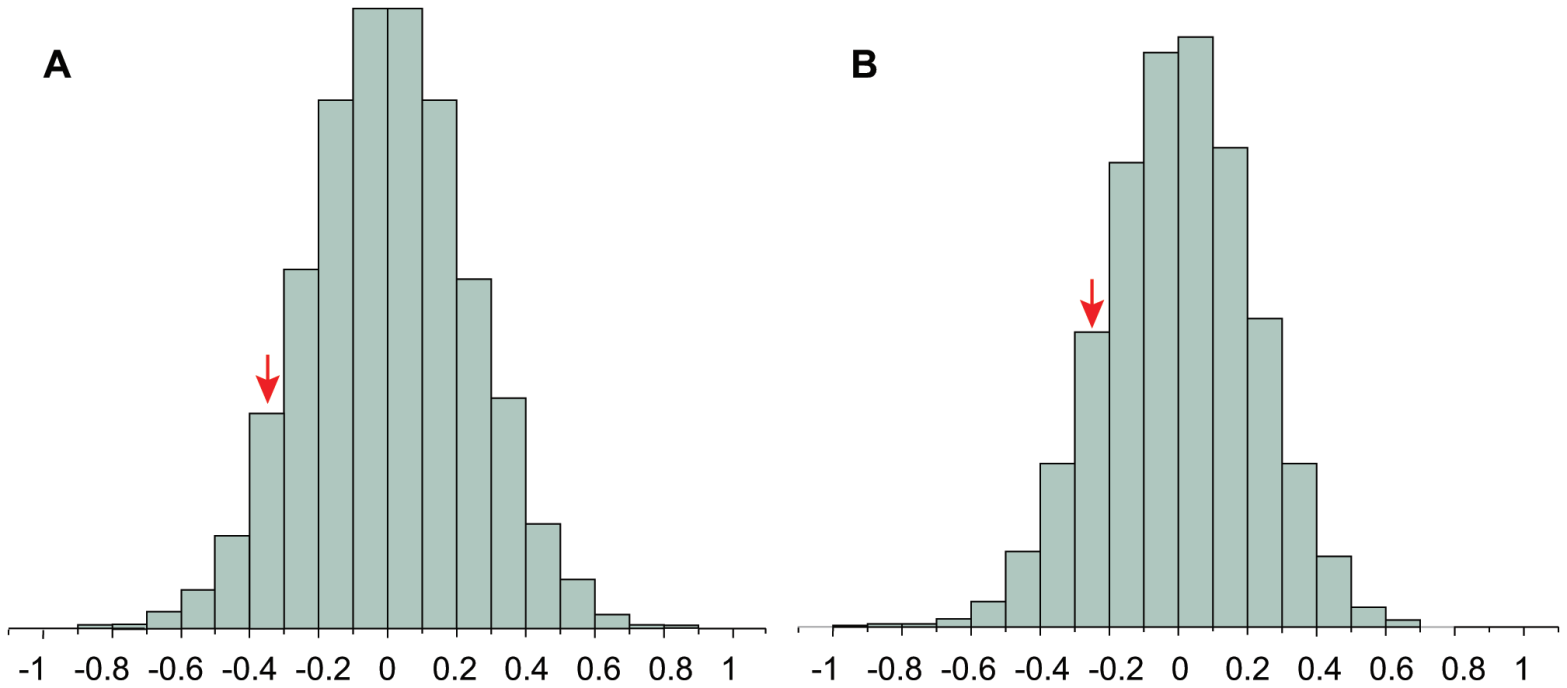
Null distributions showing range of acoustic variation in wild birds. Arrows indicate where the songs of captive-reared individuals fall within the sampling distribution generated from the songs of wild males (**A**) and females (**B**), where songs are described by PC1 (Table S1). Null distributions were generated with 10,000 bootstrap replicates.

First, we found that there was no significant difference in the structure of crystallized adult songs produced by captive-reared and wild *H. naevioides* as defined by the first three principal components (PC) extracted from song (Table 2). Second, none of the adult songs produced by captive-reared females and males grouped separately from those produced by wild birds in the DFA. In contrast, all adult songs produced by both captive-reared and wild *H. naevioides* grouped separately from those of *H. naevius* in the DFA (Table 3). Finally, the result of the bootstrap test revealed that the mean structure of captive-reared songs (as defined by PC1) did not differ significantly from that of wild songs as determined by the sampling distribution extracted from observed data on wild song structure for both males (Fig. 5A) and females (Fig. 5B; *P*>0.1 in all cases). Although our experimental sample (N = 5) is small, we note that our wild population is far better sampled (N = 32), and thus our bootstrapping procedure is able to assess the statistical significance of a relatively small number of observations, in line with previous studies (e.g. [41]).

**Table 3:** Discriminant function analysis grouping (proportion) of song profiles by captive-reared no tutor and heterospecific treatment individuals with respect to song profiles by wild conspecific *H. navioides* and heterospecific wild *H. naevius*.

No Tutor	Heterospecific Tutor	Wild *H. naevioides*	Wild *H. naevius*	Total correct
0	0	1	1	0.9

Wilks lambda	*F*	Df num	Df den	*P*
0.11	9.56	15	114	<0.0001

Our analyses reveal a high degree of underlying structural similarity between adult captive-reared and wild *H. naevioides*, but we note that one individual in the heterospecific tutor group developed songs with apparent anomalous peaks in the second note of each syllable (see Fig. 1E). Given that this structural feature is reminiscent of the songs of wild *H. naevius* (Fig. 1H), we cannot rule out the possibility that subtle or marginal vocal learning occurs in at least some *Hylophylax* individuals.

## Discussion

We have shown that *H. naevioides* individuals raised in captivity under experimental conditions produced crystallized adult songs that were statistically indistinguishable from those produced by wild adults. The same result was obtained for individuals raised with no tutor as those with a heterospecifc tutor. These results indicate that normal adult songs of *H. naevioides* develop in the absence of a conspecific tutor, thus providing the first experimental evidence that tracheophone suboscines can develop songs without learning. By this we mean that songs appear to develop without the need to encode tutor song, and we do not rule out other forms of learning, such as honing the use of vocal motor-control systems (i.e. sensorimotor learning) [42]. Given that study individuals could hear themselves singing, sensorimotor learning may have influenced the rate of progression to adult song in our experiments, but it cannot explain accurate song development in captive-reared individuals lacking conspecific tutors.

Our findings are consistent with a number of anecdotal observations from tracheophones over recent decades. For example, a single captive barred antshrike (*Thamnophilus doliatus*) produced normal song after being reared in silence [7], although it is not clear at what age it was taken into care. Similarly, one of the only known hybrid tracheophone suboscines was an antpitta (*Grallaria*) that produced a song structurally intermediate between the songs of its putative parent species [43]. This contrasts with the situation in hybrid oscines in which the structure of songs is typically unchanged from one or other of the parental song types, either because hybrid offspring copy songs from the parent male, or produce repertoires containing songs from both parental types (mixed singing) [44]–[46]. It has also been noted that there is little evidence of individual song variation, mimicry, repertoires or dialects in tracheophones, all suggesting an absence of vocal learning [30], [47], [48].

It is plausible that vocal learning may be the ancestral state in passerines and their sister-group, the parrots. Song learning may theoretically have a single ancient origin at the root of this parrot–passerine clade, in which case non-learning suboscines have potentially lost the ability to learn songs during their evolutionary history. This may explain why even non-learning suboscines possess rudimentary substrates for learning [2], which in turn may explain why we detect a subtle adjustment in the songs of one individual *H. naevioides* in the heterospecific tutor group (Fig. 1E). Regardless of whether suboscines are losing or evolving the ability to learn songs, the distribution of vocal learning in non-tracheophones appears to be restricted to lineages with polygamous or lek-breeding reproductive strategies, and absent from those with social monogamy, suggesting that sexual selection may promote vocal learning [5]. Our results support this idea by indicating a lack of learning in tracheophone suboscines, a group with long-term social bonds, apparently low levels of extra-pair copulations, and thus relatively weak sexual selection [49], [50]. The positive association between sexual selection and vocal learning across suboscines suggests either that elevated sexual selection promotes the evolution of vocal learning because it allows the development of repertoires known to mediate female choice in birds, or else that reduced levels of sexual selection can lead to the loss of vocal learning over time.

It is worth noting that the ability to learn song is retained in many oscine systems with social monogamy, and that in these cases its function is not exclusively sexual. For example, vocal learning has been shown to facilitate territory defence within species through the matching of song repertoires by neighbours [51], [52] and may also drive convergence between species when territorial signals mediate interspecific competition [53]. Although it is clear that learning can function in these agonistic contexts, such contexts do not evidently predict song learning in suboscines. So far, evidence for learning in suboscines is restricted to systems without territoriality (e.g. lek-breeding systems), whereas learning is absent in systems with intense territoriality within and between species [17], [31]. Thus, the evidence from suboscines suggests that song learning is more likely to arise from mechanisms of sexual selection (e.g. mate choice and intrasexual competition for access to matings) than from non-sexual forms of social selection (e.g. inter- and intrasexual competition for resources such as food or territories).

Our demonstration of reduced or negligible vocal learning in a tracheophone suboscine supports the increasing focus on this clade as a study system for testing evolutionary theory [18], [27], [54]. Until recently, most studies of birdsong were focused on oscine songbirds, which are potentially unsuitable subjects for some key questions because of vocal learning. For example, habitat-related differences in oscine song are often proposed to result from acoustic adaptation to habitat features [55]–[57] whereas an alternative explanation is that song differences are entirely caused by young individuals learning the songs, or parts of songs, that they perceive most clearly in their natal habitat [55]. Relationships between oscine song structure and environmental variables may therefore lack a genetic basis, and be driven instead by phenotypic plasticity [58]. Likewise, the convergence of oscine song in contact zones has been attributed to convergent character displacement [59], yet this could be explained by heterospecific copying, where one species accidentally learns the song, or song types, of heterospecifics within the contact zone [45]. Our results therefore add weight to the argument that studies focusing on tracheophone suboscine songs can overcome these problems because non-learning increases our confidence that song variation has a genetic basis [17], [27], [60].

Captive rearing experiments also provide insights into song development after hatching in tracheophone suboscines. All five study individuals followed a similar ontogeny of song development, with an initial babbling stage followed by a subsong stage where various notes and segments of adult song were produced, leading eventually to the production of incipient song and, finally, crystallized adult song. This sequence is similar to that described in several species of oscine [61]. Progress to the first crystallized adult song was relatively rapid and consistent, taking 3–4 months in all but one individual (Fig. 2). This period was longer than that reported in *Empidonax alnorum*, which can produce a rudimentary form of adult song immediately after fledging the nest [7], and shorter than that reported for *Sayornis phoebe*, which has a prolonged period (7–8 months) of incipient song until crystallization is reached at the beginning of the breeding season [2]. Both these species are non-tracheophone suboscines with simple, innate songs. Production of the first crystallized song in *H. naevioides* do not appear to be timed to the breeding season, with all birds producing adult song at least 2 months prior to the typical nesting period of wild birds. This matches observations in *Hypocnemis* antbirds, where 3-month-old juveniles begin to sing with their parents to defend family territories outside the breeding season (J. Tobias and N. Seddon, *unpublished data*).

We have shown that *H. naevioides*, a socially monogamous suboscine species with long-term pair bonds, is capable of developing normal adult songs when raised in silence or with heterospecific tutors. In all cases the songs of captive-reared individuals were not significantly different from the songs of wild adults, although we do show evidence that a small amount of imprinting on heterospecific tutors may occur. Taken together, these findings support the view that vocal learning is absent or negligible in the tracheophone suboscine clade, confirming its importance as a model system to address questions in ecology and evolution, including the nature of selection driving signal evolution and speciation. Our results also strengthen support for the hypothesis that the evolution or retention of vocal learning is associated with sexual selection in suboscines, perhaps providing clues for the origin of vocal learning mechanisms more generally.

## Supporting Information

Table S1 Factor loadings of acoustic measures for four sets of Principal Components Analyses
(DOCX)
