# Supplementary material for: Captive Rearing Experiments Confirm Song Development without Learning in a Tracheophone Suboscine Bird
Source: PLoS One. 2014 Apr 30;9(4):e95746. doi: 10.1371/journal.pone.0095746 (PMC4005748; doi:10.1371/journal.pone.0095746)
Supplement: Table S1 — Factor loadings of acoustic measures for four sets of Principal Components Analyses (DOCX) [file pone.0095746.s001.docx]

**Table S1.** Factor loadings of acoustic measures for four sets of Principal Components Analyses

|  | ***H. naevioides*** | | | ***H. naevioides*** | | | ***H. naevioides*** | | | **All species** | | |
| --- | --- | --- | --- | --- | --- | --- | --- | --- | --- | --- | --- | --- |
|  | **(sexes pooled)** | | | **(males only)** | | | **(females only)** | | |  |  |  |
|  | PC1 | PC2 | PC3 | PC1 | PC2 | PC3 | PC1 | PC2 | PC3 | PC1 | PC2 | PC3 |
| Eigenvalue (Standard Deviation) | 3.13 | 2.36 | 1.03 | 3.08 | 2.5 | 1.16 | 3.08 | 2.46 | 1.14 | 2.82 | 2.54 | 1.60 |
| % Variance | 0.52 | 0.29 | 0.06 | 0.50 | 0.33 | 0.07 | 0.50 | 0.32 | 0.07 | 0.42 | 0.34 | 0.13 |
| *Acoustic parameters* |  |  |  |  |  |  |  |  |  |  |  |  |
| Centre Frequency (Hz) |  |  |  |  |  |  |  |  |  |  |  |  |
| Entire Song | –0.30 | 0.06 | –0.16 | –0.30 | 0.04 | 0.22 | 0.31 | –0.02 | –0.01 | 0.34 | 0.00 | 0.01 |
| 1st Half Song | –0.30 | 0.13 | 0.00 | –0.30 | 0.13 | 0.12 | 0.32 | –0.07 | –0.09 | 0.32 | 0.02 | 0.22 |
| 2nd Half Song | –0.28 | 0.00 | –0.16 | –0.30 | 0.02 | –0.12 | 0.28 | 0.06 | 0.18 | 0.31 | 0.03 | –0.18 |
| Long Note* | –0.30 | 0.12 | 0.03 | –0.30 | 0.12 | –0.10 | 0.32 | –0.05 | 0.01 | 0.31 | –0.01 | 0.28 |
| Short Note* | –0.29 | 0.18 | –0.04 | –0.30 | 0.10 | 0.04 | 0.31 | –0.08 | –0.05 | 0.26 | 0.19 | –0.26 |
| Delta 1st/2nd half of song | –0.19 | 0.21 | 0.16 | –0.13 | 0.20 | 0.37 | 0.24 | –0.15 | –0.28 | 0.17 | 0.00 | 0.45 |
| 1st Quartile Frequency (Hz) |  |  |  |  |  |  |  |  |  |  |  |  |
| Long Note* | –0.30 | 0.12 | 0.00 | –0.31 | 0.12 | –0.05 | 0.31 | –0.05 | –0.04 | 0.30 | –0.04 | 0.30 |
| Short Note* | –0.29 | 0.11 | –0.05 | –0.29 | 0.08 | –0.00 | 0.31 | –0.10 | –0.01 | 0.27 | 0.16 | –0.26 |
| 3rd Quartile Frequency (Hz) |  |  |  |  |  |  |  |  |  |  |  |  |
| Long Note* | –0.30 | 0.11 | 0.05 | –0.30 | 0.12 | –0.15 | 0.31 | –0.04 | 0.06 | 0.31 | 0.01 | 0.27 |
| Short Note* | –0.29 | 0.14 | –0.01 | –0.28 | 0.14 | 0.05 | 0.31 | –0.08 | –0.05 | 0.26 | 0.21 | –0.24 |
| Inter-Quartile Bandwidth (Hz) |  |  |  |  |  |  |  |  |  |  |  |  |
| Long Note* | –0.14 | –0.00 | 0.30 | –0.17 | 0.10 | –0.55 | 0.09 | 0.08 | 0.56 | 0.18 | 0.15 | 0.04 |
| Short Note* | –0.19 | 0.15 | 0.12 | –0.13 | 0.30 | 0.25 | 0.21 | –0.02 | –0.11 | 0.18 | 0.24 | –0.15 |
| Centre Time (sec) |  |  |  |  |  |  |  |  |  |  |  |  |
| Long Note* | 0.15 | 0.37 | –0.02 | 0.15 | 0.35 | 0.00 | –0.08 | –0.39 | 0.02 | –0.12 | 0.36 | 0.11 |
| Short Note* | 0.15 | 0.37 | –0.05 | 0.17 | 0.34 | 0.04 | –0.07 | –0.40 | 0.05 | –0.10 | 0.37 | 0.09 |
| 1st Quartile Time (sec) |  |  |  |  |  |  |  |  |  |  |  |  |
| Long Note* | 0.15 | 0.37 | –0.04 | 0.15 | 0.35 | 0.01 | –0.07 | –0.39 | 0.04 | –0.11 | 0.37 | 0.10 |
| Short Note* | 0.15 | 0.37 | –0.05 | 0.16 | 0.34 | 0.04 | –0.07 | –0.40 | 0.06 | –0.10 | 0.37 | 0.09 |
| 3rd Quartile Time (sec) | 0.15 | 0.37 | –0.00 | 0.15 | 0.35 | –0.00 | –0.08 | –0.40 | 0.01 | –0.12 | 0.36 | 0.13 |
| Short Note* | 0.15 | 0.37 | –0.04 | 0.17 | 0.34 | 0.04 | –0.07 | –0.40 | 0.05 | –0.10 | 0.37 | 0.08 |
| Inter-Quartile Duration (sec) |  |  |  |  |  |  |  |  |  |  |  |  |
| Long Note* | 0.03 | 0.04 | 0.90 | 0.04 | 0.22 | –0.62 | –0.09 | 0.03 | –0.74 | –0.16 | –0.08 | 0.45 |

*Denotes mean was calculated for these notes within a song
